# Celecoxib inhibits insulin-like growth factor 1 induced growth and invasion in non-small cell lung cancer

**DOI:** 10.3892/ol.2013.1277

**Published:** 2013-03-29

**Authors:** CHEN-HUI LIU, HONG-GUANG BAO, YA-LI GE, SHU-KUI WANG, YAN SHEN, LI XU

**Affiliations:** 1 Departments of Anesthesiology, Affiliated Nanjing Hospital of Nanjing Medical University, Nanjing 210006, P.R. China; 2 Laboratory Medicine, Affiliated Nanjing Hospital of Nanjing Medical University, Nanjing 210006, P.R. China.

**Keywords:** celecoxib, non-small cell lung cancer, insulin-like growth factor axis, p-AKT

## Abstract

Despite a large number of studies indicating that celecoxib plays an important role in the prevention and treatment of tumors, the detailed molecular mechanisms are not well understood. The aim of the present study was to investigate the effect of celecoxib on insulin-like growth factor 1 (IGF-1)-induced growth and invasion in non-small cell lung cancer (NSCLC). For these experiments, IGF-1-induced cell growth and invasion were analyzed in A549 cells in the presence and absence of celecoxib. The effects of celecoxib on the expression of phosphorylated type-1 IGF receptor (IGF-1R) and phosphorylated AKT (p-AKT) were examined using western blot analysis. The influence of celecoxib on IGF-binding protein-3 (IGFBP-3) expression was analyzed using ELISA. Celecoxib inhibited IGF-1-stimulated growth and invasion in a dose-dependent manner. Celecoxib also reduced the expression of IGF-1R, IGFBP-3 and phosphorylation of AKT. The results suggest that modulating the IGF axis may be a new mechanism for the anticancer effect of celecoxib on NSCLC.

## Introduction

Although the prognosis of non-small cell lung cancer (NSCLC) has been improved by several advancements in its diagnosis and treatment, more effective and novel strategies for therapies and prevention need to be developed. Non-steroidal anti-inflammatory drugs (NSAIDs) have a chemopreventive effect. NSAIDs inhibit two COX isoforms, COX-1 and COX-2. Inhibition of COX-2 activity is thought to be the primary mechanism by which NSAIDs exert their antitumor effects (1). It has been reported that COX-2 is overexpressed in NSCLC (2). Tumor cells with elevated levels of COX-2 are highly apoptosis-resistant, angiogenic, invasive and suppressive of host immunity (3). The highly selective COX-2 inhibitors are thought to be a preferable chemopreventive agent for NSCLC. Despite epidemiological and experimental evidence indicating an important role for the use of celecoxib, a highly selective COX-2 inhibitor, in the treatment and prevention of NSCLC (4,5), the exact mechanism remains unclear. Conversely, however, recent studies have shown that even the highly selective COX-2 inhibitors have potential side effects (6). These agents should, therefore, be used at a low dosage.

The insulin-like growth factor (IGF) axis is an important growth-regulatory pathway that is prevalent in a variety of cancer types, including NSCLC. The IGF axis is composed of ligands IGF-1 and -2, their receptors, IGF-binding proteins (IGFBPs) and other regulatory factors. The IGF axis is activated by the IGFs via the type 1 IGF receptor (IGF-1R) and it is also inhibited by IGFBPs via a variety of IGF-dependent or -independent ways (7,8). The complex balance between IGFs and IGFBP-3 determines the outcome for tumor cells between survival, growth or death. Abundant data garnered from clinical studies have confirmed that increased serum IGF-1 and/or decreased IGFBP-3 levels are risk factors for growth, invasion and metastasis of many malignancies, including NSCLC (9), colon (10) and breast cancer (11). In order to prove that increased serum IGF-1 levels are a risk factor, studies have shown that systemic recombinant human IGF-1 (rhIGF-1) may stimulate the growth of tumors directly by stimulating mitosis in athymic mice (12), while the reduced circulating IGF-1 levels delayed the onset of chemical and genetic factors in induced mouse mammary tumors (13). Another study showed that IGFBP-3 may play a role in tumorigenesis and that IGFBP-3 levels could be used in future in cancer risk assessment/prevention or as markers of response to cancer treatments (14).

IGF-1R, a protein tyrosine-kinase cell surface receptor (two extracellular 125-KDa α-subunits and two transmembrane 95 KDa β-subunits). Like COX-2, IGF-1R mediates many cellular processes, including proliferation, survival and metabolism (15). IGF-1R expression is independently related to the outcomes of patients with NSCLC. Overexpression of IGF-1R may be a useful predictor of lymph node metastasis, recurrence and post-surgical outcomes in patients with NSCLC (16). Under normal conditions, the binding of IGFs to IGF-1R leads to activation of downstream signaling pathways, such as the phosphatidylinositol 3′-kinase/AKT-kinase (PI3K/AKT) signaling pathway, and increasing proliferation and survival (17).

As previously described, NSCLC cells frequently harbor high levels of COX-2 and PI3K/AKT. The antitumor effect of celecoxib partially depends on PI3K/AKT and the function of the IGF axis is closely related with the PI3K/AKT signaling pathway. As these factors appear to be so similar in their signaling mechanisms, it raises the possibility that the IGF axis may be involved in the anticancer effect of celecoxib on NSCLC. In the present study, the effects of celecoxib on IGF-1-induced growth and invasion in A549 cells were investigated. To clarify the underlying mechanism of action, the effects of celecoxib, especially at a low dosage, on the expression of phosphorylated IGF-1R and IGFBP-3 were examined. Whether the AKT signaling pathway is involved in the antitumor effect of celecoxib was examined.

## Materials and methods

### Cells and culture

Non-small cell lung cancer A549 cells were purchased from The Chinese Academy of Science, Shanghai Cell Preservation Center, China. The cells were cultured at 37°C with 5% CO_2_ in RPMI-1640 medium containing 10% fetal bovine serum (FBS). The cells were subcultured every 2–3 days and cells in the logarithmic growth phase were used. This study was approved by the ethics committee of The Affiliated Nanjing Hospital of Nanjing Medical University. Informed consent was obtained from all patients.

### Drugs and reagents

Celecoxib was purchased from Pfizer Inc. (New York, NY, USA). IGF-1 was obtained from Pepro Tech (London, UK). FBS, RPMI-1640 medium, DMSO and penicillin-streptomycin were obtained from Gibco BRL (Gaithersburg, MD, USA). The rabbit anti-IGF-1R β-subunit polyclonal antibody (anti-IGF-1R/ phospho-Tyr1131) and rabbit anti-human p-AKT polyclonal antibody were purchased from Santa Cruz Biotechnology Inc. (Santa Cruz, CA, USA). The anti-phosphorylated AKT antibody was purchased from Cell Signaling Technology (Beverly, MA, USA).

### Cell growth assay

Approximately 2×10^4^ cells per well were seeded into 96-well plates with 10% FBS and RPMI-1640 and cultured for 48 h. The cells were washed twice with PBS and cultured with 0.5% FBS and RPMI-1640 for 3 days. After serum starvation, the cells were treated with different doses of IGF-1 for 4 days. Treatment with different concentrations of celecoxib was started 24 h after the IGF-1 treatment was started and continued for 5 days. The medium was changed every day. After 4-day treatment, the net numbers of viable cells were detected using a water-soluble tetrazolium [2-(2-methoxy-4-nitrophenyl)-3-(4-nitrophenyl)-5-(2,4-disulfophenyl)-2*H*-tetrazolium, monosodium salt] colorimetric assay. The cell growth rate was calculated as the ratio of absorbance on day 4 to that on day 0.

### Cell invasion assay

To determine the invasion potential of A549 cells with/without celecoxib, in addition to IGF-1, an invasion assay was carried out, using a BD BioCoat Matrigel invasion chamber (Becton Dickinson, Frankin Lakes, NJ, USA). Cells were cultured with different concentrations of celecoxib for 36 h. The cells (2×10^4^) were then plated with 0.5% FBS RPMI-1640 in the upper chamber, containing the same dose of celecoxib. The lower chamber was filled with the same medium and IGF-1 was added as a chemoattractant. After another 12 h incubation with 5% CO_2_ at 37°C, the cells in the upper chamber were removed. The cells invading through the filter were manually counted.

### Western blot analysis

The cells were trypsinized and ∼1.5×10^5^ cells were plated in 4.0 ml of 10% FBS and RPMI-1640 in T25 flasks for 48 h. At this 48 h time point, the cells were washed twice with PBS. The celecoxib treatment at low dosage (12.5 *μ*mol/l) and/or IGF-1 (40 ng/ml) was started at plating. For the western blot analysis, the cells were washed twice with PBS, lysed in ice-cold lysis buffer [50 mmol/l Tris-HCl (PH 7.4), 150 mmol/l NaCl, 1% NP40 and 0.25% sodium deoxycholate] containing protease (Complete mini; Roche, Basel, Switzerland) and phosphatase (NaF and NaVO_3_ at 1 mmol/l; Sigma-Aldrich, MO, USA). The total protein concentrations were quantified using BCA Protein Assay kit (Pierce, IL, USA). Centrifuged lysates were separated on 10% SDS-polyacrylamide gels. After electrophoresis, the proteins were transferred to nitrocellulose, the filters probed with the primary antibodies and western blotting was carried out using the electrogenerated chemiluminescence system.

### IGFBP-3 ELISA

For detection of IGFBP-3, the cells and their treatments were the same as those used for western blot analysis. The concentrations of IGFBP-3 in the supernatants were detected by IGFBP-3 Active ELISA kit (Diagnostic System Laboratories, TX, USA). Supernatants (25 *μ*l) diluted in 50 *μ*l of assay buffer were used to quantify IGFBP-3 as specified by the manufacturer.

### Statistical analysis

The results were evaluated by one-way ANOVA to detect significant differences among the treatment groups using SPSS 17.0 statistical software. P<0.05 was considered to indicate a statistically significant result.

## Results

### Celecoxib protects A549 cells against IGF-1 induced cell growth

**Figure 1:**
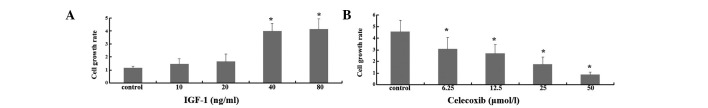
Effect of celecoxib on insulin-like growth factor-1 (IGF-1)-induced cell growth. The growth assay was carried out using the water-soluble tetrazolium colorimetric assay. The cell growth rate was calculated as the ratio of absorbance on day 4 to that on day 0. (A) Effect of different doses of IGF-1 on cell growth without celecoxib. (B) Effect of different concentrations of celecoxib on cell growth with IGF-1 (40 ng/ml). ^*^P<0.05 vs. control.

After being serum-starved with 0.5% FBS for 3 days, the A549 cells were treated with IGF-1 in the presence or absence of celecoxib. The water-soluble tetrazolium colorimetric assay was then used. The absorbance on day 4 divided by the absorbance on day 0 was defined as the growth rate. IGF-1 stimulation produced a 2-fold growth of A549 cells and the maximum effective dose of IGF-1 was ∼40 ng/ml (Fig. 1A). Celecoxib significantly protected A549 cells against IGF-1-induced cell growth in a dose-dependent manner (Fig. 1B).

### Celecoxib protects A549 cells against IGF-1-induced cell invasion

**Figure 2:**
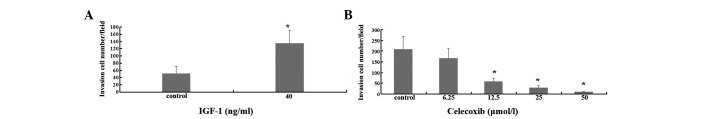
Effect of celecoxib on insulin-like growth factor-1 (IGF-1)-induced cell invasion. To determine the influence of celecoxib on IGF-1-induced cell invasion, the BioCoat Matrigel invasion chamber was used. The cells (2×10^4^) were plated with RPMI-1640 including 0.5% fetal bovine serum (FBS) in the upper chamber. The lower chamber was filled with the same medium and IGF-1 (40 ng/ml) as chemoattractant. After 12 h treatment, the cells invading through the filter were manually counted. Results were expressed as number of cells/total field (magnification, ×400). (A) Effect of IGF-1 (40 ng/ml) on cell invasion without celecoxib. (B) Effect of different concentrations of celecoxib on IGF-1 (40 ng/ml)-stimulated cell invasion. ^*^P<0.05 vs. control.

The invasion assay was carried out using a BioCoat Matrigel invasion chamber. The cells were cultured with different doses of celecoxib for 36 h. The cells were plated with 0.5% FBS, RPMI-1640 and celecoxib in the upper chamber. The same medium and IGF-1 were added to the lower chamber. The cell invasive potential was significantly (3-fold) enhanced by IGF-1 (ng/ml) as compared with the control (Fig. 2A). Consistent with the results of the cell growth assay, celecoxib significantly inhibited A549 cells against IGF-1-induced cell invasion (Fig. 2B).

### Celecoxib suppresses phosphorylation of IGF-1R

**Figure 3:**
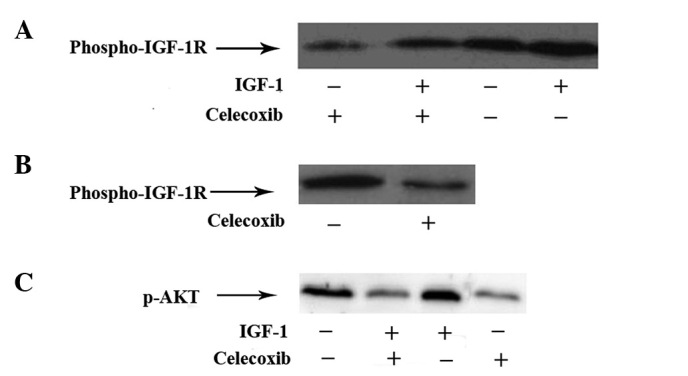
Western blot results of phosphorylated insulin-like growth factor-1R (IGF-1R) and p-AKT expression in A549 cells after exposure to IGF-1, celecoxib or a combination of these two agents. IGF-1 was used at 40 ng/ml and celecoxib was used at 12.5 *μ*mol/l. (A) Effect of IGF-1, celecoxib or combination of these two agents on the expression of phosphorylated IGF-1R. Cells (∼1.5×10^5^) were plated in 4.0 ml of 10% fetal bovine serum (FBS) and RPMI-1640 in T25 flasks for 48 h. The celecoxib treatment and/or IGF-1 was started at plating. The cell lysates were collected. Phosphorylated IGF-1R expression was analyzed using western blot analysis. (B) Effect of celecoxib on the expression of phosphorylated IGF-1R. The celecoxib treatment was the same as described in Fig. 3A. (C) Effect of IGF-1, celecoxib or combination of these two agents on the expression of p-AKT.

It was postulated that celecoxib protected A549 cells against IGF-1-induced cell growth and invasion via the IGF-1R pathway. Furthermore, phosphorylation of the triple tyrosine cluster Tyr1131/1135/1136 in the intracellular kinase domain of IGF-IR is caused by autophosphorylation and is required for activation of the intracellular kinase domain of IGF-IR (3). The phosphorylation of Tyr1131 of IGF-1R in A549 cells by western blot analysis was investigated. As shown in Fig. 3A, celecoxib reversed the IGF-1 activated phosphorylation of IGF-1R. The pIGF-1R-Tyr1131 level was decreased after 48 h treatment of celecoxib at low dosage (Fig. 3B).

### Celecoxib inhibits expression of p-AKT

The AKT signaling pathway plays a crucial role in proliferation, invasion and metastasis of tumor cells. AKT is the key regulatory factor of the AKT signaling pathway. p-AKT is the phosphorylated state of AKT. Only p-AKT has a biological function. The expression of p-AKT was examined using western blot analysis. As shown in Fig. 3C, IGF-1 stimulation increased expression of p-AKT. Treatment with celecoxib at low dosage (12.5 *μ*mol/l) decreased the level of p-AKT and reversed the action of IGF-1-induced phosphorylation of AKT.

### Celecoxib upregulates IGFBP-3

**Figure 4:**
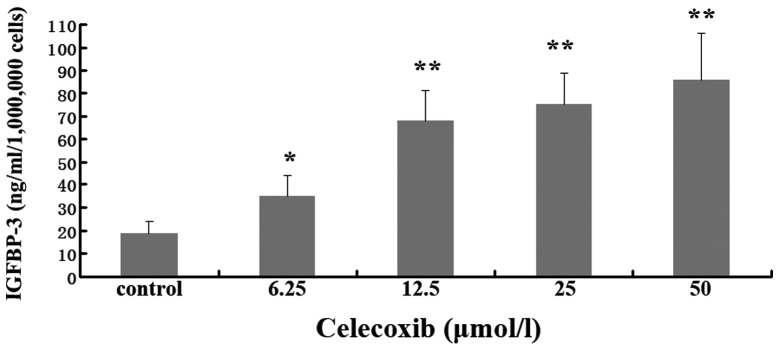
Celecoxib increased insulin-like growth factor binding protein 3 (IGFBP-3) expression. The IGFBP-3 concentrations in supernatants were detected by IGFBP-3 active enzyme-linked immunosorbant assay (ELISA) kit. ^*^P<0.05 vs. control; ^**^P<0.01 vs. control.

The influence of celecoxib on the expression of IGFBP-3 was detected using ELISA. As shown in Fig. 4, exposure of A549 cells to celecoxib significantly elevated the levels of IGFBP-3 in tumor cell culture supernatants, even when the dose of celecoxib was very low (6.25 *μ*mol).

## Discussion

Lung cancer is a malignant tumor with a high incidence worldwide, and NSCLC accounts for 75–80% of cases. The five-year survival rate has not exceeded 15% in the past few decades (18). Surgical excision remains the mainstay of treatment for NSCLC. Paradoxically, the perioperative period represents a high risk of tumor cell metastasis (19). Celecoxib, a highly selective COX-2 inhibitor, is commonly administered to patients in order to relieve postoperative pain. One study demonstrated that celecoxib suppresses cancer progression, in addition to its analgesic and anti-inflammatory effect (20). Furthermore, it has been shown that perioperative COX-2 inhibition reduces the adhesion and metastatic potential of disseminating tumors in murine models and improves cancer recurrence-free survival rates in mice undergoing primary tumor excision (21). Celecoxib may exert its antitumor effect via COX-2-dependent and -independent pathways.

Despite many studies indicating that celecoxib plays an important role in the prevention and treatment of tumors, the detailed molecular mechanisms are not well understood. It was postulated that there is a functional association between COX-2 inhibitors and the IGF axis in NSCLC. These could be attributed to three possible mechanisms.

The first is that celecoxib suppresses phosphorylation of IGF-1R. The results of the present study indicate that celecoxib downregulates the expression of phosphorylated IGF-1R (Fig. 3A). The IGF axis plays an important role in tumor growth, invasion and metastasis. IGF-1 is a potent mitogen for both normal and tumor cells. Vlachostergios *et al* measured the baseline IGF-1 plasma levels in 77 patients who were diagnosed with metastatic NSCLC. Their results showed that IGF-1 was correlated with systemic inflammation and appeared to play an independent predictive role in metastatic NSCLC (22). The present study confirms that IGF-1, a major ligand for IGF-1R, has an important role in the growth and invasion in NSCLC cells (Fig. 1A and 2A). The function of IGF-1 is mediated primarily by the IGF-1R. As previously described, IGF-1R is a heterotetramer containing two α-and two β-subunits. Binding of the ligand (IGFs) to the α-subunit triggers a conformational change that leads to the autophosphorylation of a triple tyrosine cluster Tyr1131/1135/1136 of the intracellular kinase domain in the β-subunit (3,23). Autophosphorylation strongly enhances the activity of the IGF-1R catalytic domain. Activation of IGF-IR upregulates PI3K/AKT signaling and increases proliferation and survival. Combined with previous findings, this suggests that in future NSCLC targeted therapies, COX-2 and IGF-1R inhibitors could be combined.

The second mechanism may be that celecoxib upregulates the expression of IGFBP-3. The activities of the IGF axis are strictly regulated by a family of IGFBPs, especially IGFBP-3. IGFBP-3, a major serum carrier protein for IGFs, is a multi-functional protein known to inhibit cellular growth and induce apoptosis of various cancer cells (24). IGFBP-3 inhibits IGF-induced biological effects by binding to IGFs, thereby blocking IGF binding (25). Furthermore, overexpression of COX-2 by tumor cells downregulates IGFBP-3 mRNA expression (26). In the present study, celecoxib upregulated expression of IGFBP-3, even when the dose was low (Fig. 4). Thus, celecoxib-mediated upregulation IGFBP-3 in NSCLC cells could decrease the mitogenic and invasive potential of IGF-1.

The third possible mechanism is that celecoxib down-regulates expression of p-AKT. As previously described, AKT is a serine/threonine protein kinase also known as protein kinase B, which is one of the key pathways modulating cell growth, proliferation, metabolism, survival and angiogenesis (27). p-AKT is the activated state of AKT that is highly expressed in most tumors. Only p-AKT has biological characteristics (28). Uddin *et al* reported that inhibition of COX-2 by NS398, a highly selective COX-2 inhibitor, impaired phosphorylation of AKT, resulting in decreased downstream signaling leading to cell growth inhibition and induction of apoptosis in the epithelial ovarian carcinoma (EOC) cell line (29). Another study suggested that celecoxib exerted its antitumor activities in human osteosarcoma cell line MG-63 through COX-2-independent mechanisms, which may be PI3K/AKT-dependent. PI3K may be at the center of the celecoxib effects (30). In the current study, western blot analysis was used to determine the p-AKT protein expression in A549 cells. The results show that the p-AKT protein was decreased in A549 cells with the treatment of celecoxib (Fig. 3C). These results illustrate that in A549 cells, celecoxib-inhibited cell growth and invasion may be related to inhibition of the PI3K/AKT pathway. p-AKT downregulation is another potential target for the future prevention and treatment of NSCLC.

There were limitations in the present study. The detailed mechanisms of celecoxib on the IGF-1R and AKT signaling pathway need to be studied. It remains unclear whether a longer treatment period would improve the outcome.

**Figure 5:**
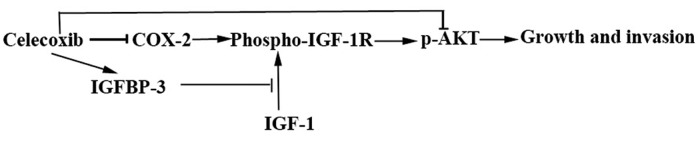
The possible mechanisms of celecoxib-induced antitumor effect. Celecoxib suppresses phosphorylation of insulin-like growth factor 1 receptor (IGF-1R), upregulates the expression of IGF binding protein 3 (IGFBP-3) and downregulates the AKT signaling pathway.

In summary, celecoxib inhibits the growth and invasion of NSCLC cells via the IGF axis and AKT pathway. Celecoxib suppresses phosphorylation of IGF-1R, upregulates the expression of IGFBP-3 and downregulates the AKT signaling pathway. This suggests a close correlation between COX-2 inhibitors, the IGF axis and the PI3K/AKT pathway (Fig. 5). It suggests that celecoxib administration as adjuvant therapy or in combination with PI3K/AKT inhibitors and/or IGF-1R inhibitors could be of therapeutic benefit for patients with NSCLC.
